# Influence of the Molecular Weight of the Polycarbonate Polyol on the Intrinsic Self-Healing at 20 °C of Polyurethanes

**DOI:** 10.3390/polym16192724

**Published:** 2024-09-26

**Authors:** Yuliet Paez-Amieva, José Miguel Martín-Martínez

**Affiliations:** Adhesion and Adhesives Laboratory, University of Alicante, 03080 Alicante, Spain; yuliet.paez@ua.es

**Keywords:** intrinsic self-healing at 20 °C, polyurethane, molecular weight of the polyol, polycarbonate polyol, tack, mechanism of self-healing

## Abstract

Different polyurethanes (PUs) were synthesized with polycarbonate polyols of molecular weights of 500, 1000, and 2000 Da. Their self-healing abilities at 20 °C were tested, and their structural, thermal, and mechanical properties were analyzed. The PUs made with polycarbonates of molecular weights 500 (YC500) and 1000 Da (YC1000) exhibited self-healing at 20 °C, and the self-healing time of YC1000 was the shortest. The absence of crystallinity and the low degree of micro-phase separation favored self-healing at 20 °C in YC500. However, the presence of tack and the existence of allophanate species and urethane–carbonate and urea–carbonate hydrogen bonds disfavored self-healing. Consequently, the self-healing time at 20 °C of YC500 was longer than expected. On the other hand, YC1000 exhibited an “equilibrium” between urethane-carbonate and urea–carbonate hydrogen bonds and carbonate–carbonate interactions among the soft segments, so a particular structural order was produced that was associated with its fastest self-healing at 20 °C. The PU made with the polycarbonate of molecular weight 2000 Da did not exhibit self-healing at 20 °C because of its significant micro-phase separation, the presence of semi-crystalline soft domains, and the lower density of hydrogen bonds.

## 1. Introduction

Polyurethanes (PUs) are commonly used in automotive, construction, furniture, textile, and biomedical industries [1,2]. During use, their lifetime is reduced by mechanical deterioration, and difficult-to-recycle chemical residues are produced that are causing environmental concerns [3,4]. Nowadays, the self-healing of PUs is a way to increase their durability [5].

PUs are synthesized by reacting isocyanate with polyol and chain extender. Most PUs exhibit a segmented structure consisting in long polyol chains (soft segments—SS) and urethane/urea linkages (hard segments—HS). The physicochemical, surface, adhesion, and mechanical properties of the PUs can be tuned depending on the synthesis conditions and the chemical nature and structure of the reactants [6]. HS and SS can crystallize, and micro-phase separation is produced due to the thermodynamic incompatibility between them [7,8,9,10,11,12].

PUs display micro-phase separation, and they can be designed to exhibit self-healing mediated by reversible hydrogen bonds. Hydrogen bonds between neighboring urethane groups may contribute to impart self-healing [13]. In addition, a high mobility of the soft segments to diffuse through cracks is necessary for imparting self-healing [14,15]. The chemical structures of HS and SS and the length of the soft segments in the PU chains determine both the intermolecular hydrogen bonds and the mobility of the soft segments [16]. In this study, the influence of the molecular weight of the polyol or SS length on the self-healing ability of PUs is mainly considered.

Different studies on the structure-property relationship in PUs made with polyols of different molecular weights have been carried out [8,17,18,19,20,21,22,23,24,25,26,27,28,29,30,31]. Klinedinst et al. [8] studied the effects of varying SS molecular weight and HS content in thermoplastic segmented PUs, and they found that the higher molecular weight of the polyol favored the partial crystallization of SS and higher micro-phase separation. In addition, Cho et al. [17] used poly (tetramethylene glycol)s—PTMGs—of molecular weights 1000 and 1800 Da and mixtures of them for synthesizing PUs. The PUs made with PTMGs showed different interactions between HS, such as hydrogen bonding, dipole-dipole, and induced dipole-dipole interactions; these interactions, significantly influenced the micro-phase separation.

Although the most common soft segments in PUs are polyethers or polyesters, several studies dealing with PUs made with poly (alkylene carbonates) soft segments have been published [18,19,20,21,22,23,24,25,26,27,28,29,30,31]. These PUs exhibit high tensile strength and elasticity [18,19,20,21,22] and good resistance to hydrolysis and organic solvents [23,24,25,26,27,28,29,30]. Liu et al. [32] synthesized waterborne polyurethanes (WPUs) with polycarbonate polyols (PCDs) of different molecular weights (1000–2000 Da), and they concluded that the decrease in the molecular weight of PCD increased the HS content and the amounts of urethane and urea groups. In addition, the increase in the molecular weight of PCD improved the thermal stability, crystallinity, and mechanical properties of the PUs. Similarly, García et al. [33] synthesized WPUs with polycarbonates of 1,6-hexanediol of different molecular weights (500–3000 Da), and they showed that the increase in the molecular weight of PCD increased the degree of micro-phase separation and decreased the HS content. On the other hand, Eceiza et al. [34] also synthesized PUs by using polycarbonates of 1,6-hexanediol of molecular weights 1000 and 2000 Da, and they found reduced ability of SS to crystallize in the PU made with PCD of 1000 Da that also exhibited a mixed phase of HS and SS. However, the PU made with PCD of 2000 Da showed significant micro-phase separation and semi-crystalline soft domains.

There are few studies in the literature on how the length of the soft segments affects the self-healing of PUs, especially those made of polycarbonates [16,35]. Liang et al. [16] obtained PUs with PCD and polypropylene glycol (PPG) blends of different molecular weights (400, 600, 1000, 2000 Da). The self-healing was ascribed to reversible hydrogen bonds and the mobility of the PPG segments. The increase in the molecular weight of PPG increases the mobility of the polymeric chains but decreases the density of hydrogen bonds, so the self-healing was reduced. On the other hand, Ha et al. [35] synthesized PUs made with PCDs of different molecular weights (1000–3000 Da) containing disulfide bonds. The PUs made with PCD blends exhibited stronger interactions between the hard segments and improved mechanical properties with respect to the PUs made with one PCD only. Furthermore, the blending of PCDs of different molecular weights and the existence of disulfide bonds resulted in stretchable and transparent PUs showing self-healing after heating at 50–70 °C for 10–60 min.

The most self-healing PUs do not show fast self-healing at room temperature. In previous study [36], the fast intrinsic self-healing at 20 °C of one PU made with polycarbonate of 1,6-hexanediol (YCD) of molecular weight 1000 Da has been demonstrated. The self-healing of this PU was attributed to a significant number of free carbonate groups, a relatively low amount of urethane groups, a low micro-phase separation, and a high mobility of the soft segments. In another study [37], different PUs made with mixtures of polyester and polycarbonate polyols of molecular weights 1000 Da have shown self-healing at 20 °C; the kinetics of self-healing were slow, and the self-healing time was longer by increasing the amount of polyester soft segments. The self-healing was ascribed to moderate micro-phase separation, reduced ability of SS to crystallize, the existence of a mixed phase of HS and SS, and the interactions between the polyester and polycarbonate soft segments.

Due to the absence of studies on the self-healing at room temperature of PUs made with a single polycarbonate polyol, in this work, different PUs made with polycarbonates of 1,6-hexanediol of different molecular weights (500, 1000, and 2000 Da) were synthesized to find out how the soft segment length influences the previously proposed intrinsic self-healing mechanism [36].

## 2. Materials and Methods

### 2.1. Materials

The PUs were synthesized by reacting 4,4′ methylene bis(cyclohexyl) isocyanate (HMDI) (90% purity, Sigma Aldrich Co., St. Luis, MO, USA) with polycarbonates of 1,6-hexanediol polyols of molecular weights 500 (CD500), 1000 (CD1000), and 2000 Da (CD2000) (UBE Chemical Europe S.A., Castellón, Spain). The prepolymer obtained was reacted with 1,4-butanediol (BD) chain extender (99% purity, Panreac Applichem^®^, Darmstadt, Germany).

#### 2.1.1. Synthesis of the Polyurethanes (PUs)

The PUs were similarly synthesized by using the one-shot method; the only difference was the molecular weight of the polycarbonate of 1,6-hexanediol: 500 Da (YC500), 1000 Da (YC1000), and 2000 Da (YC2000). An NCO/OH ratio of 1.1 was used. The required amounts of polyol (3.2051 g for CD500, 3.9062 g for CD1000, and 4.3868 g for CD2000) and 1,4-butanediol (0.0578 g for CD500, 0.035 g for CD1000, and 0.0198 g for CD2000) were placed in a polypropylene bottle heated at 80 °C. After stirring in a double centrifuge SpeedMixer DAC 150.1 FVZ-K equipment (FlackTek Inc., Landrum, SC, USA) at 2400 rpm for 1 min, the mixture was placed in an oven at 80 °C for 10 min. Afterwards, the isocyanate (1.7949 g for CD500, 1.0938 g for CD1000, and 0.6140 g for CD2000) was added to the polyol + 1,4-butanediol mixture and stirred in a SpeedMixer at 2400 rpm for 1 min. Finally, the PUs were cured in an oven for 7.5 h in cycles of 30 min at 50, 60, and 70 °C followed by heating at 80 °C. The PUs were annealed at 85 °C for 1 h.

At least three batches of each PU were synthesized (the amount of each PU batch was 5 g), and they were fully reproducible. The hard segments contents of the PUs varied between 13 (YC2000) and 37 wt.% (YC500).

#### 2.1.2. Experimental Techniques

**Figure 1:**
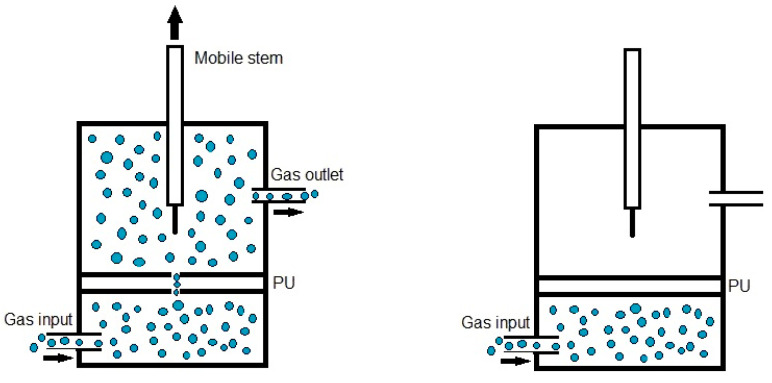
Diagram of the equipment used to assess the self-healing at 20 °C of the PUs.

Quantitative evaluation of self-healing. The self-healing at 20 °C of the PUs was determined by using the equipment described in a previous study [38]. Cylindrical PU pieces (dimensions: diameter = 19 mm; thickness = 3 mm) were hermetically placed inside a thermostatic chamber. Each PU piece was fully pierced with a needle of diameter 1 mm, and, upon removing the needle, a continuous flow of nitrogen gas at a pressure of 750 mbar and a flow rate of 8 mL/min was allowed to flow from the bottom to the top of the closed chamber (Figure 1). The gas flow was continuously monitored over time until it ceased (Figure 1), allowing the determination of the self-healing time and the kinetics of self-healing of the PUs.

Tack measurement. The tack of the PUs was evaluated at 20 °C using the probe tack test. A flat cylindrical probe with a diameter of 3 mm was placed in contact with the PU surface, and then a force of 5 N was applied for 1 s. Afterwards, the probe was pulled out at a constant rate of 10 mm/s. 

Gel Permeation Chromatography (GPC). The molecular weights of the PUs were determined by GPC. The experimental setup consisted of a pump, injector, column, and detector. Water Styragel HR5 and HR3 columns (Agilent Technologies Spain, S.L., Las Rozas, Spain), a Waters 1515 isocratic high-performance liquid chromatography pump, and a Waters 2414 refractive index detector were used. For the analysis, 2 mg of solid PU was dissolved in HPLC-grade THF (tetrahydrofuran), and the solution was eluted in HPLC-grade THF (flow rate = 1 mL/min). For calibration, polystyrene standards were used. Two experiments were conducted per sample, and the results were averaged.

Differential Scanning Calorimetry (DSC). The structure and degree of micro-phase separation of the PUs were assessed by DSC. The analysis was conducted under nitrogen atmosphere (flow rate: 100 mL/min) in a DSC Q100 (TA Instruments, New Castle, DE, USA). Three consecutive thermal runs were performed: (i) heating from −80 °C to 200 °C at a heating rate of 10 °C/min; (ii) cooling from 200 °C to −80 °C at a cooling rate of 10 °C/min; and (iii) heating from −80 °C to 250 °C at a heating rate of 10 °C/min.

Wide-angle X-ray diffraction (XRD). The crystallinities of the PUs were assessed in a Bruker D8-Advance (Bruker, Etlinger, Germany) provided with a Kristalloflex K 760-80F X-ray generator (3000 W; 20–60 kV; 5–80 mA) with a copper anode. PUs with geometrical dimensions of 2 mm diameter and 0.5 mm thick and a parallel beam geometry for thin films were used. The X-ray diffractograms were normalized to the maximum peak using Evaluation 14.0.0.0 (2017) software.

Infrared spectroscopy in attenuated total reflectance mode (ATR-IR spectroscopy). ATR-IR spectroscopy was employed to analyze the chemical composition and structure of the PUs. An Alpha spectrometer (Bruker Optik GmbH, Ettlinger, Germany) equipped with a germanium prism was used. The incident angle of the infrared beam was 45°, and 60 scans were performed at a resolution of 4 cm^−1^.

X-ray photoelectron spectroscopy (XPS). K-Alpha equipment (Thermo Scientific, Waltham, MA, USA) was used to analyze the chemical composition of the PU surfaces. All spectra were collected using Al kα radiation (1486.6 eV) at 3 mA and 12 kV, and a twin crystal monochromator was used. The spot size was 400 µm, and an alpha hemispherical analyzer operated in the constant energy mode was used. Survey scan pass energy of 200 eV was carried out, and high-resolution spectra were recorded by using scan pass energies of 50 eV.

Thermal gravimetric analysis (TGA). The thermal properties of the PUs were evaluated by TGA Q500 (TA Instruments, New Castle, DE, USA) under nitrogen atmosphere (flow rate: 50 mL/min). For each test, a 9–10 mg sample was heated from 35 °C to 600 °C (heating rate = 10 °C/min).

Dynamic mechanical thermal analysis (DMA). The rheological properties and viscoelasticity of the PUs were assessed by DMA Q800 (TA Instruments, New Castle, DE, USA). The measurements were conducted in single cantilever mode, and rectangular samples of dimensions 33 mm × 12 mm × 2.5 mm were used. The samples were heated from −100 °C to 80 °C at a heating rate of 5 °C/min, and a frequency of 1 Hz was used.

Mechanical properties. Standardized type 2 dumbbell PU specimens were prepared, and stress-strain tests were carried out. The ASTM D 638 standard was selected, and the tests were carried out in a Zwick/Roell Z005 universal testing machine (Barcelona, Spain) by using a pulling rate of 100 mm/min and smooth pneumatic jaws. Three replicates were obtained for each PU, and the results were averaged.

## 3. Results

In our previous study [36], the intrinsic self-healing at 20 °C of one PU made with polycarbonate polyol of molecular weight 1000 Da was ascribed to moderate micro-phase separation, reduced ability of SS to crystallize, the existence of a mixed phase of HS and SS, and the interactions between the polycarbonate soft segments. A mechanism of intrinsic self-healing based on the existence of dynamic non-covalent exchange interactions between polycarbonate soft segments was proposed. Thus, upon damage of the PU and, due to the mobility of the soft segments, the interactions among initially bonded carbonate groups and the free carbonate groups in the soft segments were produced, causing a fast intrinsic self-healing.

Considering that the mobility of the soft segments and the number of carbonate groups differ depending on the molecular weight of the polycarbonate polyol, in this study the self-healing at 20 °C of PUs made with polycarbonates of different molecular weights was assessed.

**Figure 2:**
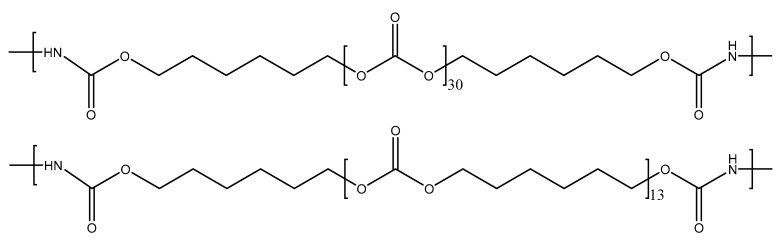
Chemical structure of the soft segments in the YC2000.

As a representative example, Figure 2 displays the number of carbonate groups and carbonate of 1,6-hexanediol units in the soft segments of YC2000. The increase in the molecular weight of the polycarbonate polyol decreases the hard segments (HS) content of the PUs from 37 wt.% to 13 wt.% (Table 1) [32]. This trend can be expected by considering that the number of carbonate of 1,6-hexanediol units and the number of carbonate groups increase from 5 to 30 in the soft segments of the PUs by increasing the molecular weight of the polyol from 500 Da to 2000 Da, respectively (Table 1). As a consequence, the mobility of the PU chains would be increased by increasing the length of the polycarbonate soft segments. However, the literature does not agree on the influence of the increase in the length of the soft segments on the self-healing ability of the PUs. Whereas Liang et al. [16] found reduced self-healing in PUs made with polycarbonate and polyether polyols by increasing the molecular weight of the polyol due to lower density of hydrogen bonds, Ha et al. [35] found the opposite in PUs made with polycarbonate polyols and disulfide moieties. Thus, depending on the number of free carbonate groups and dipole carbonate–carbonate interactions between the polycarbonate soft segments and their length, different interactions between the hard and soft segments may limit the mobility of the polymeric chains, and, thus, different self-healing abilities of the PUs can be expected.

The segmental motion of the PUs is tightly related to their molecular weights and HS contents [32,35]. The average number molecular weights (M_n_) of the PUs increase by increasing the molecular weight of the polycarbonate polyol (Table 2), and they are within the range of other self-healing PUs [35], so they should exhibit an adequate segmental mobility. However, the average molecular weights of all PUs are within the same range. On the other hand, the M_z_ values and polydispersity indexes of the PUs decrease by increasing the molecular weight of the polycarbonate (Table 2), so YC2000 has the narrowest molecular weight distribution and YC500 the widest one.

**Figure 3:**
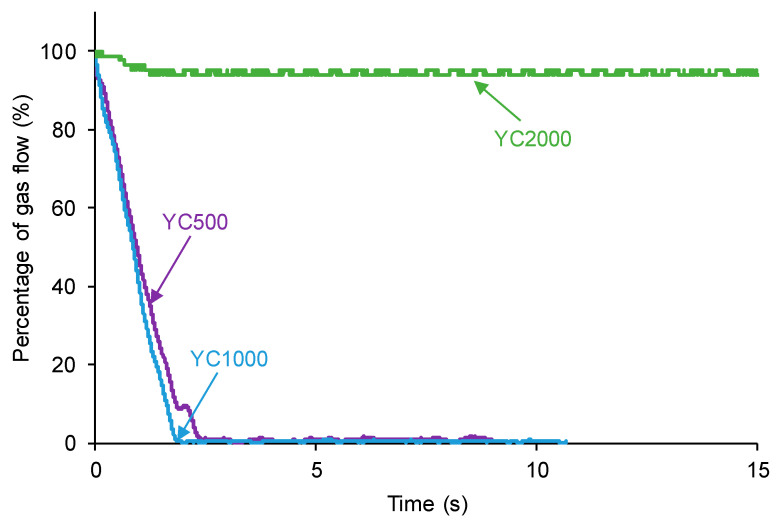
Kinetics of self-healing at 20 °C of PUs made with polycarbonates of different molecular weights.

The self-healing of the PUs made with polycarbonate polyols can be affected by their different HS contents, distinct length of the polycarbonate soft segments, and the number of free carbonate groups and carbonate–carbonate interactions between the soft segments. The kinetics of self-healing at 20 °C of the PUs are shown in Figure 3, in which it is evidenced that YC500 and YC1000 show fast self-healing (2.5 and 1.4 s, respectively) and that YC2000 does not exhibit self-healing at 20 °C. Therefore, the low hard segment content, the increase in the molecular weight, and the increase in the number of carbonate groups/carbonate of 1,6-hexanediol units do not favor PU self-healing. On the other hand, Figure 3 shows that the self-healing time of YC500 is lower (2.5 s) than the one of YC1000 (1.4 s). Considering the differences in HS content, the length of the polycarbonate soft segments, and the molecular weights, faster kinetics and shorter self-healing time can be expected in YC500 than in YC1000. 

According to Figure 3, the kinetics of self-healing of YC500 and YC1000 are fast. The curves of Figure 3 were adjusted to first-order kinetics—Equation (1):

(1) $$Rate=k\left[gas\;flow\right]$$

The plot of the gas flow vs. time allows the calculation of the rate constants of the kinetics of self-healing at 20 °C of YC500 and YC1000—Equation (2):

(2) $$\ln\left[gas\;flow\right]=-kt+ln{\left[gas\;flow\right]}_{o}$$

**Figure 4:**
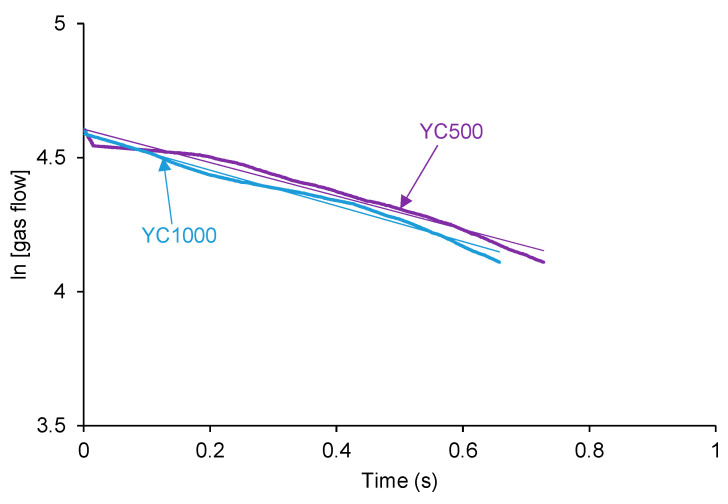
Adjustment of the kinetics of self-healing at 20 °C of YC500 and YC1000 to first order kinetics.

Figure 4 shows that the plots of ln[gas flow] vs. time adjust reasonably the kinetics of self-healing of YC500 and YC1000 to a first order (R^2^ = 0.97–0.98). The rate constant of YC500 is 0.62, and the one of YC1000 is 0.67. Therefore, the kinetics of self-healing are somewhat similar but slightly higher in YC1000.

**Figure 5:**
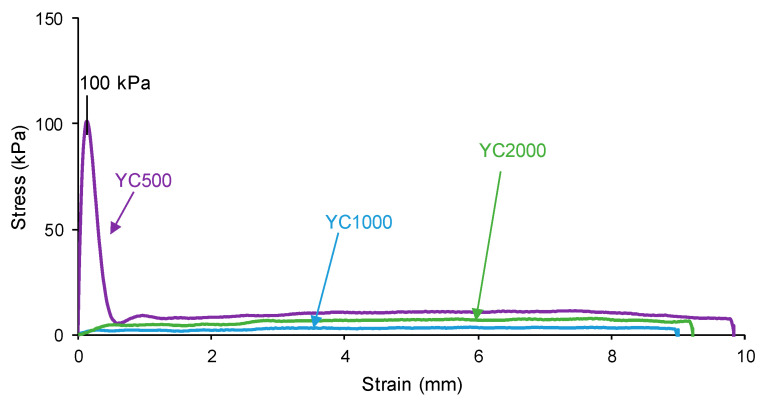
Variation of the stress at 20 °C as a function of the strain for PUs made with polycarbonates of different molecular weights. Probe tack test.

The self-healing of the PUs can be affected by the existence of tack—i.e., a stitch that holds two pieces of material together temporarily. The probe tack is the most common procedure to determine the tack of the polymers. Figure 5 shows that YC500 is the only one exhibiting tack (100 kPa) and is located at the peak of the stress–strain curve. Tack is caused by the mobility of the short chains in YC500 that will contribute to delay the self-healing because the polymeric chains will tend to displace opposing to the interactions among them. Thus, the self-healing time is longer in YC500 than in YC1000. However, some additional factors should influence the self-healing at 20 °C of YC500 and YC1000.

**Figure 6:**
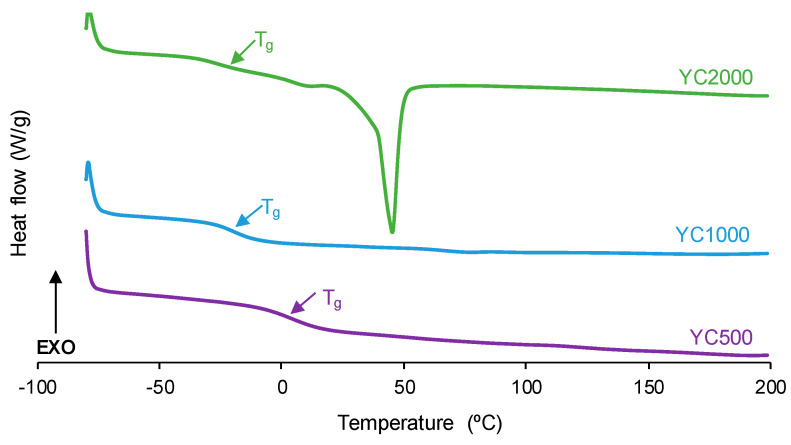
DSC curves of PUs made with polycarbonates of different molecular weights. First heating run.

The degree of micro-phase separation determines the self-healing of the PUs, and it was assessed by DSC. The DSC curves of the first heating run of the PUs show the glass transition temperature (T_g_) of the soft segments, which decreases by increasing the polycarbonate molecular weight (Figure 6, Table 3). The decrease in T_g_ is expected by decreasing the hard segment content in the PUs [32]. The interactions between the soft segments determine the heat capacity at constant pressure (∆c_p_) values, which decrease by increasing the polycarbonate molecular weight. Thus, the interactions between the polycarbonate soft segments are stronger in YC500 and less important in YC2000. On the other hand, only YC2000 shows a small cold crystallization at 20 °C and the melting of the soft segments at 45 °C (melting enthalpy: 23 J/g), so the movement of the polymeric chains is restricted. Consequently, it cannot be expected that YC2000 exhibits self-healing at 20 °C.

Once the thermal history of the PUs is removed by cooling down to −80 °C, a second DSC heating run was carried out (Figure S1 of the Supplementary Materials file). The DSC curves show the glass transitions of the soft segments (T_ss_) and the hard segments (T_hs_). Whereas T_hs_ values are somewhat similar in all PUs (236–240 °C), the T_ss_ values decrease by increasing the polycarbonate molecular weight (Table 4). Thus, considering the differences between T_ss_ and T_hs_ values, YC2000 shows the greatest micro-phase separation and YC500 the lowest. The lower micro-phase separation favors the self-healing ability at 20 °C of the PUs [36], so the self-healing will be favored in the PUs made with polycarbonates of lower molecular weights (YC500 and YC1000). In a different study [35], similar conclusions were obtained as an increase in micro-phase separation was obtained by increasing the length of the polycarbonate soft segments in PUs made with polycarbonate polyols and disulfide moiety. Furthermore, in that study, the self-healing ability assessed by heating at 50–70 °C for 10–60 min was favored when low micro-phase separation was observed in the PUs.

**Figure 7:**
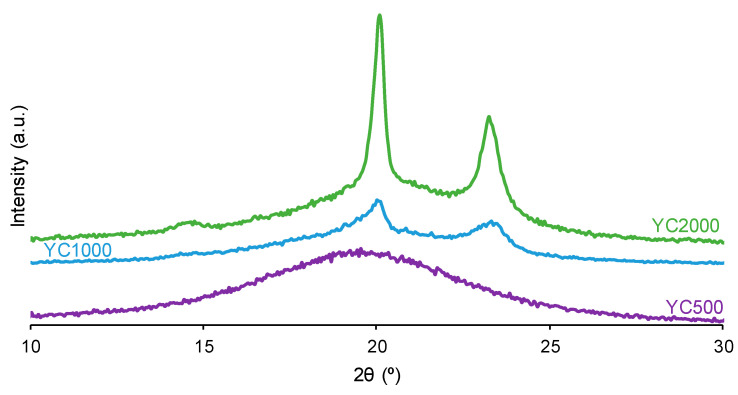
X-ray diffractograms of PUs made with polycarbonates of different molecular weights.

The crystallinity of the soft segments and the micro-phase separation affect the PU self-healing ability. Figure 7 shows that the X-ray diffractograms of YC1000 and YC2000 exhibit the characteristic diffraction peaks at 2θ = 20.0–20.1° and 2θ = 23.3° due to interactions between polycarbonate soft segments [35,37]. The intensities of the two diffraction peaks are significantly higher in YC2000 than in YC1000 (Figure 7, Table S1 of Supplementary Materials file), i.e., YC2000 exhibits higher crystallinity. On the other hand, the X-ray diffractogram of YC500 shows a wide, shallow peak typical of an amorphous structure, and the intensity of the peak is higher than the corresponding one in YC1000. The absence of crystallinity in YC500 contributes to its self-healing, and the important crystallinity of YC2000 favors poor self-healing. Because YC1000 shows the shorter self-healing time among the three PUs and exhibits some crystallinity, the interactions between the polycarbonate soft segments could play an important role in its self-healing ability. In fact, the soft segments of YC1000 have 13 carbonate groups and 6 carbonate of 1,6-hexanediol units, so the creation of net carbonate–carbonate interactions is feasible. However, the soft segments of YC500 are short as they have 3 carbonate of 1,6-hexanediol units and 5 carbonate groups only, so they should have fewer carbonate–carbonate interactions that are needed for exhibiting dynamic non-covalent interactions between the polycarbonate soft segments (proposed mechanism of self-healing). Therefore, the higher number of carbonate–carbonate interactions in YC1000, together with its reduced crystallinity, may contribute to its faster self-healing as compared to the one in YC500.

**Figure 8:**
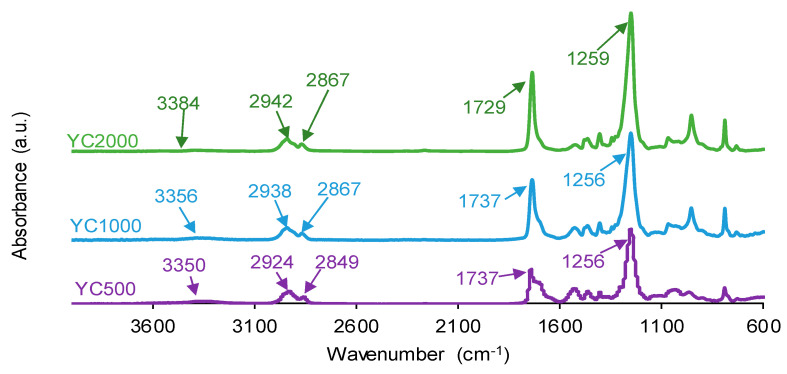
ATR-IR spectra of PUs made with polycarbonates of different molecular weights.

The chemistries of the PUs synthesized with polycarbonates of different molecular weights were assessed by ATR-IR spectroscopy. The ATR-IR spectra of all PUs show the same absorption bands (Figure 8), mainly N-H stretching band at 3350–3384 cm^−1^, C=O stretching band of urethane and urea groups at 1729–1737 cm^−1^, C-H stretching bands at 2924–2942 and 2849–2867 cm^−1^, and C-O stretching of carbonate groups at 1256–1259 cm^−1^. The increase in the molecular weight of the polycarbonate displaces the carbonyl band in the ATR-IR spectra of the PUs to lower wavenumber; furthermore, the shape of the carbonyl band varies depending on the molecular weight of the polycarbonate. These changes can be ascribed to the existence of different free and bonded (hydrogen bond, dipole carbonate–carbonate bond) C=O species in the PUs synthesized with polycarbonates of different molecular weights.

**Figure 9:**
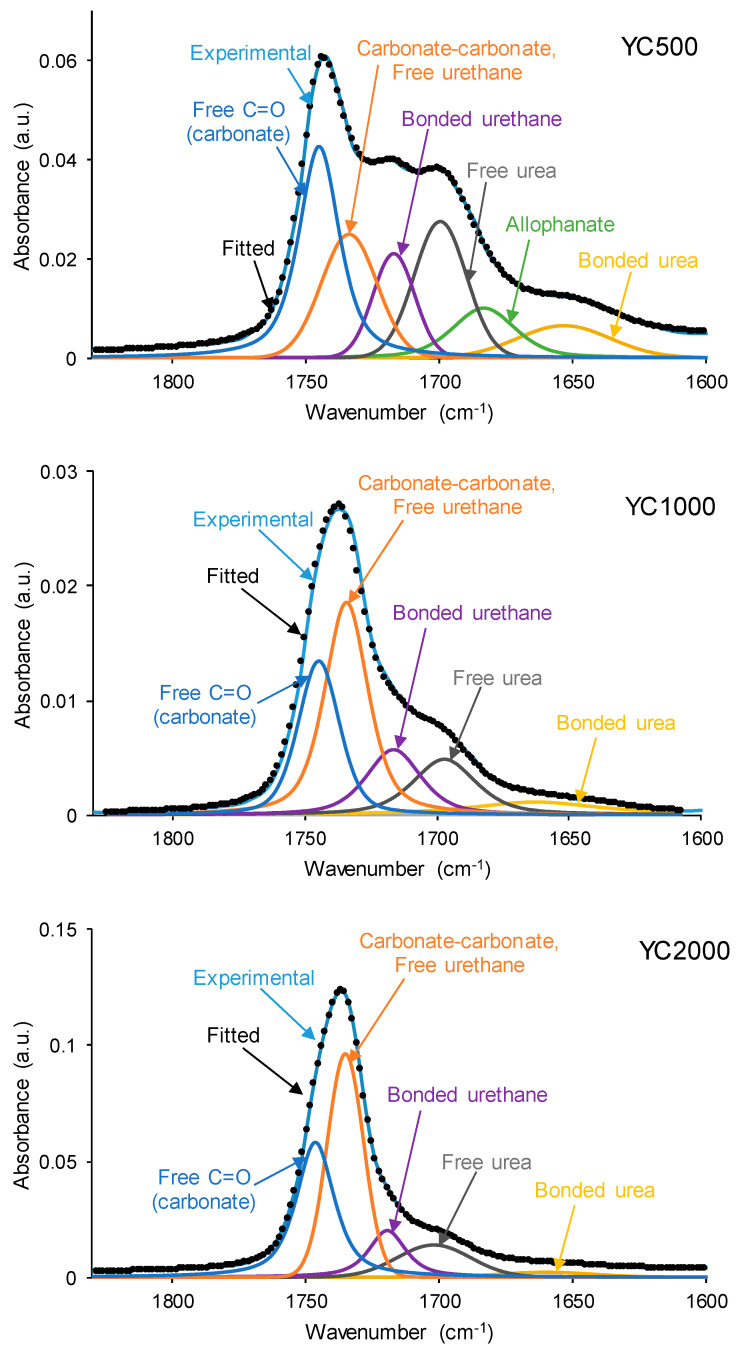
Curve fitting of the carbonyl stretching region of the ATR-IR spectra of PUs made with polycarbonates of different molecular weights.

The different C=O species in the PUs were assessed by curve fitting (a Gaussian function was used) of the carbonyl stretching bands in the ATR-IR spectra (Figure 9). All PUs show the existence of five different C=O species (Table 5) and, additionally, only YC500 exhibits allophanate groups at 1685 cm^−1^ [39]. The allophanate groups are produced by the reaction of urethane and isocyanate and only appear in YC500 because of the short polyol chain. The existence of allophanate groups inhibits the segmental motion of the polymer chains, and this can be associated with longer self-healing time in YC500. All PUs have 24–34% free carbonate groups at 1742–1746 cm^−1^ [40], and YC1000 has the lowest percentage. Furthermore, the curve fitting of all PUs shows free urethane and dipole carbonate–carbonate interactions at 1732–1733 cm^−1^ [40,41], and its percentage increases from YC500 to YC1000. The hydrogen-bonded urethane species appear at 1713–1722 cm^−1^, and its percentage is somewhat similar in all PUs. Additionally, the free and hydrogen-bonded urea groups appear at 1695–1697 and 1650–1654 cm^−1^, respectively [42], and their percentages decrease by increasing the molecular weight of the polycarbonate. Therefore, YC500 shows a significant percentage of free carbonate and free and hydrogen-bonded urea groups, a low percentage of urethane groups, and is the only one having allophanate species (Table 5). On the other hand, YC1000 shows a low percentage of free carbonate and significant percentages of free urethane/dipole carbonate–carbonate interactions and bonded urea species (Table 5). Considering the different C=O species, YC500 shows allophanate species that increase the interactions between its polymeric chains, whereas YC1000 has a lower percentage of free carbonate and a higher percentage of dipole carbonate–carbonate interactions, and these different chemical species may contribute to the longer self-healing time of YC500 with respect to YC1000.

The chemical species on the PU surfaces were also assessed by XPS. Survey XPS spectra show that the PU surfaces contain 76–85 at.% carbon, 14.9–23.6 at.% oxygen, and 0.1–3% nitrogen (Table 6). In general, the atomic percentage of carbon increases and the one of oxygen decreases by increasing the molecular weight of the polycarbonate soft segments in the PUs. However, almost similar carbon percentages (76–78 at.%) are obtained in YC500 and YC1000, but the oxygen content is higher in YC1000; in fact, YC1000 exhibits the highest oxygen content among the three PU surfaces. Therefore, the chemical structures of YC500 and YC1000 are different, in agreement with the evidence provided by the ATR-IR spectra. Furthermore, because of the lower hard segment content in YC2000, the atomic carbon content is the highest and the one of oxygen is the lowest among the three PU surfaces. On the other hand, the high-resolution N1s spectra of the PU surfaces show one contribution only at a binding energy of 399.9–400.1 eV due to -NH-COO- species of urethane/urea. Because the hard segment content of the PUs decreases from YC500 to YC2000, the atomic nitrogen percentage decreases by increasing the molecular weight of the polycarbonate soft segments.

**Figure 10:**
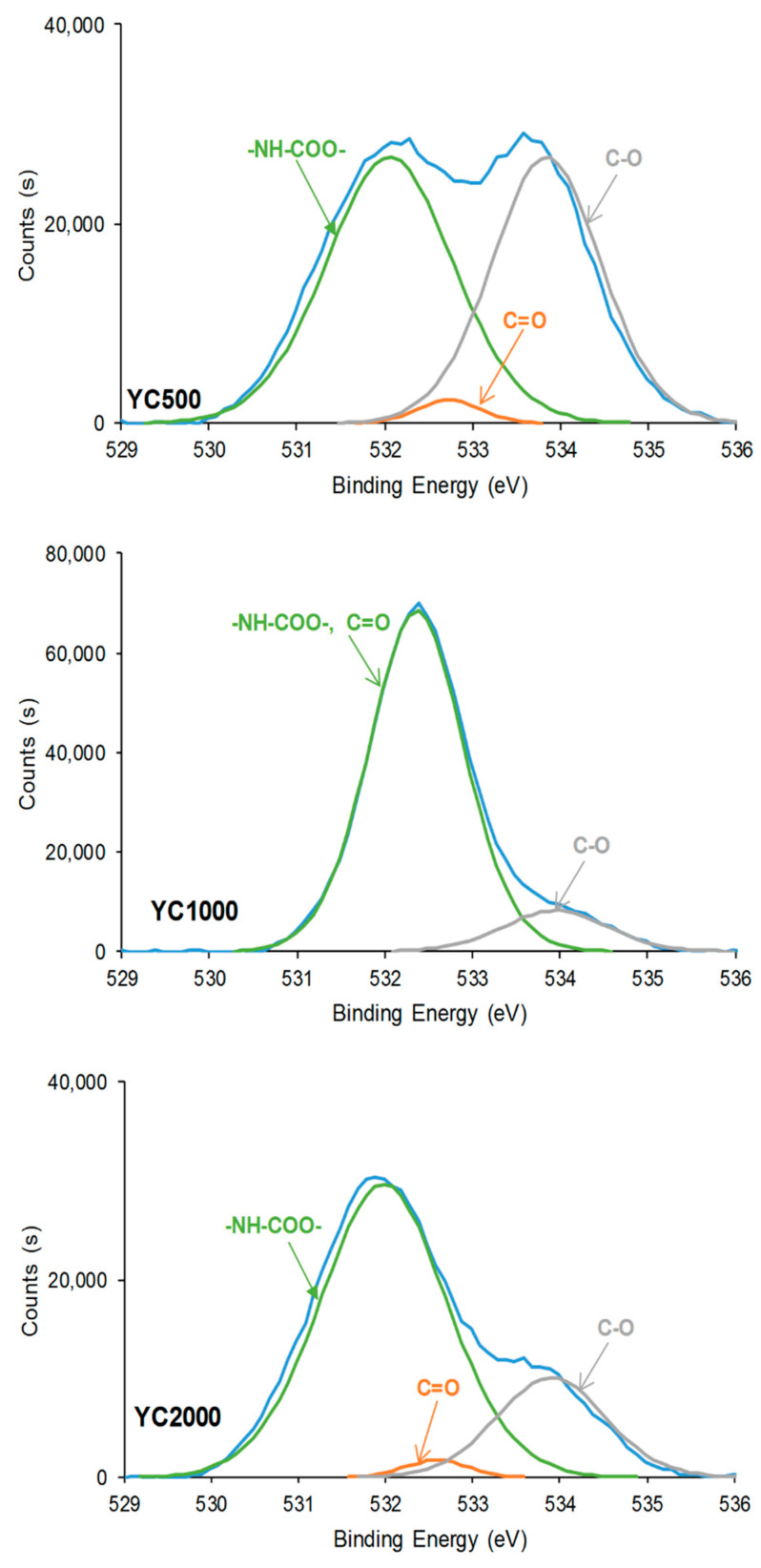
High resolution O1s XPS spectra on the surfaces of PUs made with polycarbonates of different molecular weights.

In general, the curve fittings of the high-resolution O1s photopeaks of the PU surfaces show three species: -NH-COO-, C=O, and C-O (Table 7). -NH-COO- species at binding energies of 531.9–532.1 eV (Figure 10) are dominant in all PUs. In fact, in a previous study, the binding energy of –NH-COO- of PU surfaces appeared at 530.8 eV [36]. However, the lower binding energy of the band in the O1s photopeak of YC1000 (532.4 eV) is intermediate between the one of -NH-COO- (531.9–532.1 eV) and C=O species (532.6–532.7 eV). Therefore, in YC1000, the –NH-COO- and C=O species are difficult to distinguish, likely due to its relatively low free C=O species and high dipole carbonate–carbonate interactions (they are evidenced by ATR-IR spectroscopy). In fact, YC1000 exhibits the highest percentage of –NH-COO-/C=O species and the lower one of C-O species (Table 7), and this is an indication of its different structure with respect to YC500 and YC2000. This agrees well with the results of the curve fitting of the carbonyl region of the ATR-IR spectra.

Because the hard segment content decreases from YC500 to YC2000, a decrease in the percentage of -NH-COO-, C=O, and C-O species can be expected as the length of the polycarbonate soft segments increases. However, according to Table 7, the percentages of -NH-COO- and C=O species are higher in YC2000 than in YC500. This can be attributed to the existence of allophanate groups in YC500 and the higher percentage of free C=O groups in YC500 and YC2000, in agreement with the curve fitting of the carbonyl region of the ATR-IR spectra.

**Figure 11:**
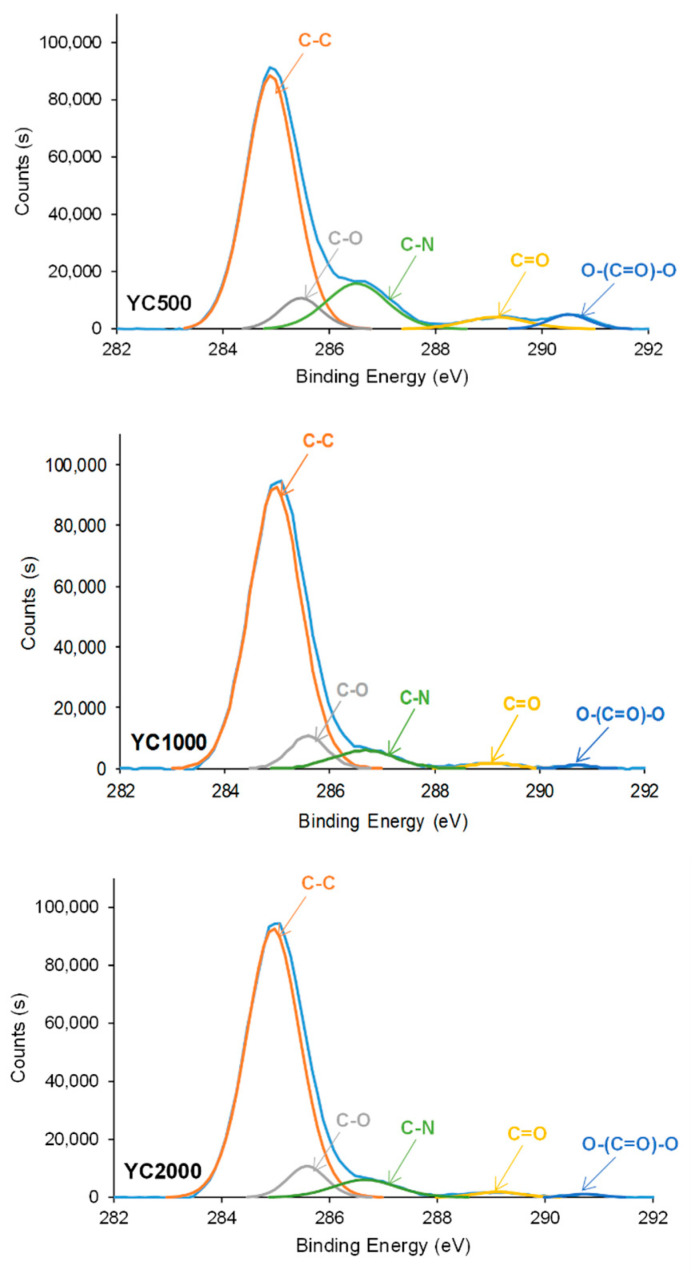
High resolution C1s XPS spectra on the surfaces of PUs made with polycarbonates of different molecular weights.

The curve fittings of the high resolution C1s photopeaks on PU surfaces show the existence of C-C (binding energy = 284.9 eV), C-O (binding energy = 285.6–285.7 eV), C-N (binding energy = 286.7–286.8 eV), C=O (binding energy = 289.1–289.4 eV), and O-(C=O)-O (binding energy = 290.7–290.8 eV) species (Figure 11, Table 8). This assignment was based on a previous study [43]. The increase in the molecular weight of the polycarbonate soft segments causes an increase in the percentage of C-C species and a decrease in the ones of C-N—because of the decrease in the hard segment content—and O-(C=O)-O species. The YC1000 surface shows a higher percentage of C-C and a lower percentage of C=O species than the other PUs, due to the existence of a lower percentage of free carbonate groups and a higher percentage of dipole carbonate–carbonate interactions. On the other hand, YC500 shows a significantly high C-N percentage, likely due to allophanate species [44].

**Figure 12:**
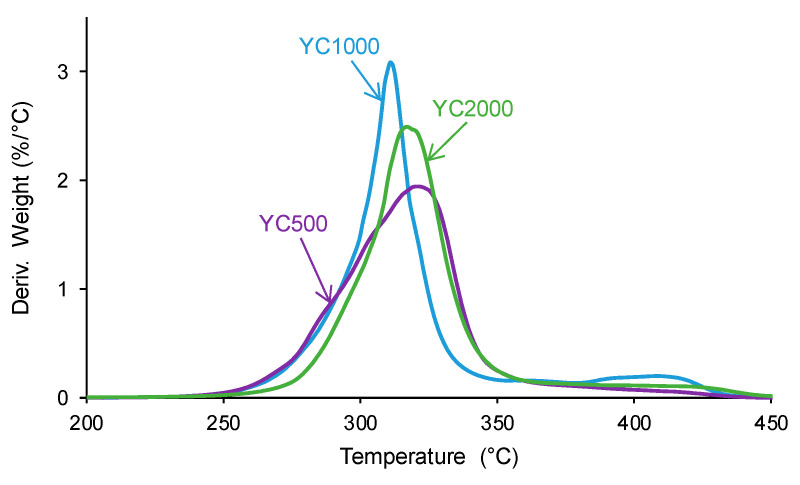
Derivatives of the TGA curves of PUs made with polycarbonates of different molecular weights.

The structural differences between the PUs were also evidenced by TGA. The TGA curves of the PUs (Figure S2 of the Supplementary Materials file) show a different trend in YC1000 with respect to the other PUs. In fact, below 300 °C, similar TGA curves are obtained in YC500 and YC1000, and, above 340 °C, the TGA curve of YC1000 is above the others. The different thermal events can be better distinguished in the derivatives of the TGA curves. Figure 12 and Table 9 show that the lowest temperature of maximum thermal decomposition corresponds to YC1000. All PUs show three thermal decompositions at 285–292 °C (carbonate–carbonate interactions [36]), 311–321 °C (mixed phase due to carbonate–urethane interactions [36,45]), and 412–421 °C (hard segments). The DTGA curve of YC500 shows the lowest temperature of thermal degradation of the carbonate–carbonate interactions and the highest temperature and weight loss of the mixed phase, in agreement with the DSC results. The DTGA curve of YC1000 shows the lowest degradation temperatures of the mixed phase and hard segments, so the mixed phase due to carbonate–urethane interactions decomposes at a lower temperature than in YC500, i.e., the movement of the polymeric chains in YC1000 is more favored than in YC500. This can be associated with the higher time of self-healing in YC500. On the other hand, the DTGA curve of YC2000 shows the highest thermal degradation temperatures.

**Figure 13:**
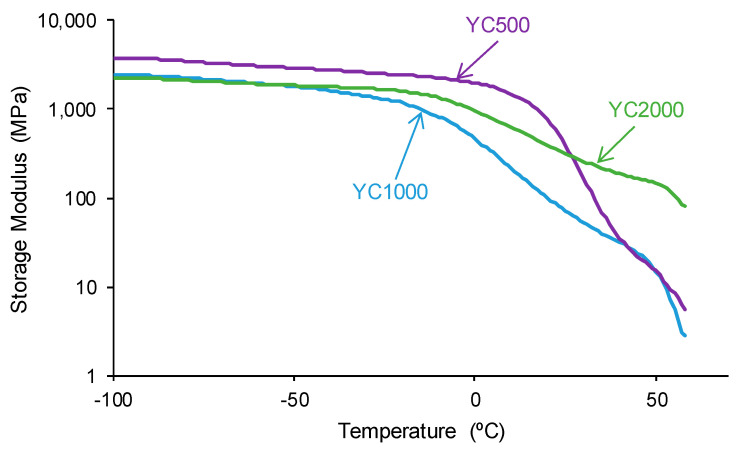
Variation of the storage (E′) moduli of PUs made with polycarbonates of different molecular weights as a function of the temperature. DMA experiments.

The structural differences between the PUs made with polycarbonates of different molecular weights affect their viscoelastic properties. The variation of the storage modulus as a function of the temperature (Figure 13) shows higher storage moduli in the glassy region in YC500 than in YC1000 and YC2000. Once the glass transition is reached, a decline of the storage moduli is produced, and the decrease is more marked in YC500 and YC1000. and less pronounced in YC2000. Thus, the movement of the polymeric chains is more favored in YC500 and YC1000 than in YC2000, and this may contribute to the existence of self-healing. This is in agreement with the results provided by DSC and TGA.

**Figure 14:**
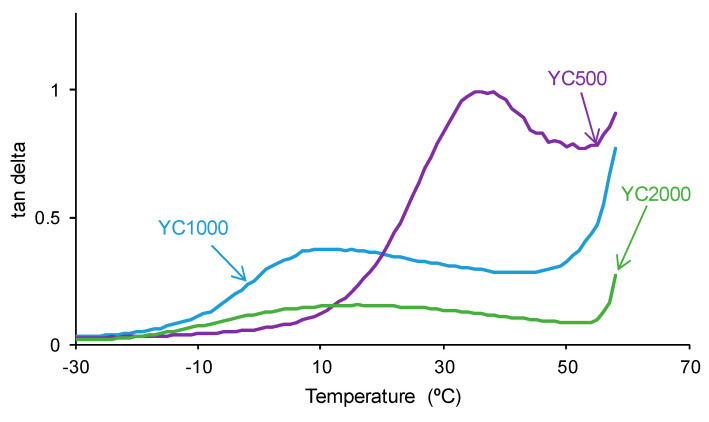
Variation of tan delta as a function of the temperature for PUs made with polycarbonates of different molecular weights as a function of the temperature. DMA experiments.

The tan delta vs. temperature plots of the PUs (Figure 14) show one structural relaxation only. YC500 shows the highest tan delta value and temperature of the structural relaxation, indicating a marked rheological viscous regime and, therefore, good polymeric chain mobility (Table 10). YC1000 shows an intermediate tan delta value and the lowest temperature of the structural relaxation, and the viscous rheological contribution is important. However, YC2000 shows a low tan delta value and dominant elastic rheological regime that supports the absence of self-healing.

**Figure 15:**
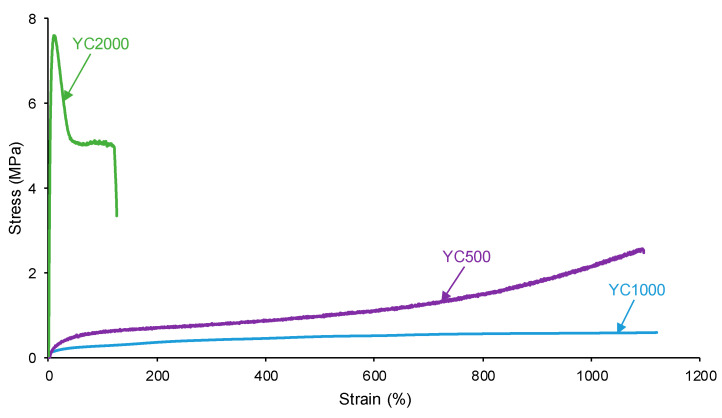
Stress–strain curves of PUs made with polycarbonates of different molecular weights.

The self-healing PUs should exhibit adequate mechanical properties. The stress–strain curves of the PUs are different (Figure 15). YC2000 shows the stress–strain curve of a stiff thermoplastic material, i.e., high tensile strength (5.0 MPa) and low elongation-at-break (119%) (Table 11). However, YC500 and YC1000 show typical stress–strain curves of elastomeric materials. Interestingly, the mechanical properties of YC1000 are lower than the ones of YC500, and this can be associated with higher mobility of the polymeric chains. The high mobility of YC1000 is related to the lower free carbonate groups and higher dipole carbonate–carbonate interaction between the soft segments with respect to YC500 and YC2000. In a different study [35], PUs made with polycarbonate polyols of molecular weights of 1000 to 3000 Da and disulfide groups were synthesized and characterized. The elongation-at-break of the PUs increased by increasing the length of the polycarbonate soft segments and the breaking stress increased, and this is in agreement with the higher mobility of the polymeric chains. However, the most relevant interactions in these PUs were tiol-disulfide, and the interactions between the polycarbonate soft segments and the urethane/urea groups were less relevant than in the PUs synthesized in this study.

## 4. Discussion

In this study, the self-healing ability at 20 °C of three PUs synthesized with polycarbonate soft segments of different lengths was assessed, and the self-healing–structure property relationship was addressed. Whereas YC500 and YC1000 show self-healing at 20 °C, YC2000 does not. Because the three PUs were similarly synthesized, the different self-healing behavior should be ascribed to the different length of the soft segments and to the extent of interactions between them.

In a previous study [36], the mechanism of intrinsic self-healing at 20 °C of one PU made with polycarbonate polyol was assigned to dynamic non-covalent exchange interactions between the polycarbonate soft segments. The features that support this mechanism were the existence of free carbonate groups and net carbonate–carbonate interactions, the reduced ability of SS to crystallize, the existence of a mixed phase of HS and SS, the high polymeric chain motion, and the low micro-phase separation between the hard and soft segments.

**Figure 16:**
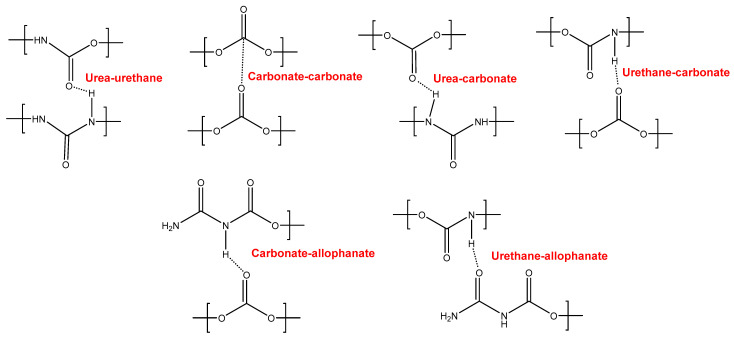
Interactions between polar groups in YC500.

YC500 exhibits a rapid self-healing at 20 °C that can be ascribed to the existence of short chains (low molecular weight, short soft segments—three carbonate of 1,6-hexanediol units and five carbonate groups) and the important hydrogen bonds among the hard segments and between urethane/urea and carbonate groups in the soft segments. However, the self-healing time is longer than in YC1000 due to the existence of tack and the high HS content in YC500, both of which reduce the mobility of the polymeric chains. Furthermore, YC500 has a high polydispersity index, so there are short polymeric chains that favor the crack closure during self-healing and also long polymer chains that should displace slowly; therefore, the formation of an ordered structure among polymer chains of different lengths would be more difficult and the interactions between the carbonate groups would be less net. On the other hand, YC500 shows a low micro-phase separation, high heat capacity at constant pressure in the glass transition of the soft segments, and lack of crystallinity. Furthermore, YC500 has allophanate species, a low percentage of carbonate–carbonate interactions, a high amount of free urethane, and free and bonded ureas, and this disfavors the interactions between the soft segments and delays the self-healing at 20 °C. Because of a significant number of free carbonate species and short polycarbonate soft segments in YC500, the formation of urea–urethane and carbonate–carbonate interactions and different hydrogen bonds (urethane–carbonate, urea–carbonate, carbonate–allophanate, and urethane–allophanate) can be expected (Figure 16), and these interactions are responsible for the high decomposition temperature of the mixed phase in the TGA experiment and the high storage moduli in the DMA experiment. 

In contrast, YC500 has high mechanical strength, high T_g_, but poor thermal property with respect to YC1000, so it could be expected to possess high thermal stability. However, due to the existence of allophanate moieties, a significant number of free carbonate species, and short polycarbonate soft segments, a different trend is found in YC500. In summary, the absence of crystallinity and the low degree of micro-phase separation favor self-healing at 20 °C in YC500. However, the presence of tack and the existence of allophanate species and urethane–carbonate, urea–carbonate, carbonate–allophanate, and urethane–allophanate hydrogen bonds disfavor self-healing. Consequently, the self-healing time at 20 °C is longer than expected for a PU with short soft segments.

**Figure 17:**
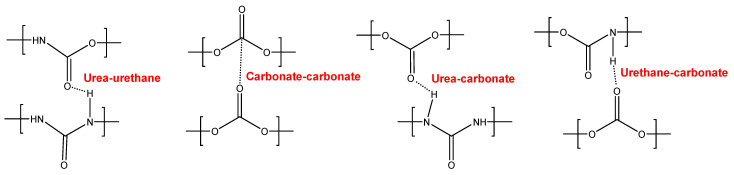
Interactions between polar groups in YC1000.

YC1000 has the fastest and most efficient self-healing at 20 °C among the three PUs of this study, and the self-healing time is shorter than in YC500. YC1000 has twice the number of carbonate groups per soft segment than YC500, and this allows the existence of some crystallinity (diffraction peaks at 2θ values of 20° and 23.3°) due to interactions between polycarbonate soft segments [35,36]. Furthermore, the micro-phase separation in YC1000 is moderate, and its heat capacity at constant pressure in the glass transition of the soft segments is significant, so the percentage of free carbonate species is relatively small and the one of carbonate–carbonate species is important. At the same time, YC1000 exhibits mobility of the soft segments because of the medium tan delta value, the lower decomposition temperature of the mixed phase in the TGA experiment, and the reduced mechanical properties (low Young´s modulus, low tensile strength, high elongation-at-break). Thus, YC1000 exhibits an “equilibrium” between the existence of urethane–carbonate, urea–carbonate and urea–urethane hydrogen bonds, and carbonate–carbonate interactions among the soft segments (Figure 17), so a substantial structural order exists that justifies the fast self-healing at 20 °C.

YC2000 does not show self-healing at 20 °C. Its low hard segment content, higher molecular weight, and the large number of carbonate groups and carbonate of 1,6-hexanediol units do not favor self-healing. Furthermore, the increase in the molecular weight of the polycarbonate polyol increases the mobility of the polymeric chains but decreases the density of hydrogen bonds, so the self-healing is inhibited [16]. On the other hand, the DSC curve of YC2000 shows cold crystallization, which is a sign of important crystallinity and a highly ordered structure of the soft phase. This was confirmed by the X-ray diffractogram of YC2000, which shows a well-defined crystalline structure with characteristic peaks at 2θ = 20.1° and 2θ = 23.3° due to the interactions between the polycarbonate soft segments. On the other hand, YC2000 shows an important micro-phase separation and a low tan delta value, which is indicative of a dominant elastic rheological behavior. Finally, YC2000 has the typical behavior of a thermoplastic stiff material. In conclusion, YC2000 presents a significant number of carbonate–carbonate interactions, free carbonate species, and hydrogen bonds between the carbonate group and the urethane; all these could favor its self-healing. However, it does not exhibit self-healing at 20 °C because it has a very ordered and rigid structure, i.e., the movement of the polymeric chains is not favored. Even more, YC2000 shows a significant micro-phase separation and crystallinity. Thus, when a rupture of YC2000 occurs at 20 °C, the carbonate–carbonate and carbonate–urethane interactions are mainly broken, and the activation energy at 20 °C is not sufficient to kinetically allow the polymeric chains to come closer and form new bonds, and it cannot self-heal.

## 5. Conclusions

The PUs synthesized with polycarbonates of molecular weights 500 and 1000 Da exhibited fast self-healing at 20 °C, but the one made with polycarbonate of molecular weight 2000 Da did not. The shorter self-healing time corresponded to YC1000 due to an equilibrium between the existence of urethane–carbonate and urea–carbonate hydrogen bonds and carbonate–carbonate interactions among the soft segments. Even though YC500 had the shorter polycarbonate soft segments and the lowest micro-phase separation, the existence of tack and the favored formation of urethane–carbonate and urea–carbonate, allophanate–carbonate, and allophanate–urethane hydrogen bonds allowed slower kinetics of self-healing and longer self-healing time than in YC1000.

YC1000 exhibited optimal self-healing at 20 °C due to moderate micro-phase separation, a relatively small percentage of free carbonate species and an important amount of carbonate–carbonate species, reduced crystallinity, and adequate mobility of the soft segments.

The absence of self-healing at 20 °C in YC2000 was due to low mobility of the polycarbonate soft segments, a high number of carbonate–carbonate interactions, significant micro-phase separation, and important crystallinity.

Finally, the mechanical properties of YC500 and YC1000 need improvement.

## Figures and Tables

**Table 1:** Number of carbonate groups and carbonate of 1,6-hexanediol units in the soft segments, and hard segments (HS) contents of PUs made with polycarbonates of different molecular weights.

PU	No. Carbonate Groups	Carbonate of 1,6-Hexanediol Units	HS (wt.%)
YC500	5	3	37
YC1000	13	6	22
YC2000	30	13	13

**Table 2:** Molecular weights and polydispersity indexes of PUs made with polycarbonates of different molecular weights.

PU	M_n_ (Da)	M_w_ (Da)	M_z_ (Da)	Polydispersity Index
YC500	13,336	58,879	182,424	4.4
YC1000	19,762	66,779	178,473	3.4
YC2000	22,739	63,729	145,734	2.8

**Table 3:** Thermal events obtained from the DSC curves of PUs made with polycarbonates of different molecular weights. First heating run.

PU	T_g_ (°C)	$\Delta c_{p}\;(J/g{}^{\circ}C)$	T_c_ (°C)	ΔH_c_ (J/g)	T_m_ (°C)	ΔH_m_ (J/g)
YC500	3	0.50	-	-	-	-
YC1000	−21	0.29	-	-	-	-
YC2000	−29	0.20	20	1	45	23

**Table 4:** Glass transition temperatures of the SS and HS of PUs made with polycarbonates of different molecular weights. Second DSC heating run.

PU	T_gss_ (°C)	T_ghs_ (°C)
YC500	9	237
YC1000	−18	236
YC2000	−36	240

**Table 5:** Percentages of different C=O species in the carbonyl stretching region of PUs made with polycarbonates of different molecular weights.

Wavenumber (cm^−1^)	Percentage (%)			Assignment
	YC500	YC1000	YC2000	
1650–1654	8	9	3	Bonded urea
1685	10	-	-	Allophanate
1695–1697	20	15	12	Free urea
1713–1722	12	14	11	Bonded urethane
1732–1733	19	38	40	Carbonate–carbonate, free urethane
1742–1746	31	24	34	Free C=O (carbonate)

**Table 6:** Chemical species on the surfaces of PUs made with polycarbonates of different molecular weights. XPS experiments.

PU	C (at.%)	O (at.%)	N (at.%)
YC500	78.0	19.0	3.0
YC1000	76.0	23.6	0.4
YC2000	85.0	14.9	0.1

**Table 7:** Binding energies and percentages of O1s species on the surfaces of PUs made with polycarbonates of different molecular weights. XPS experiments.

Species	Percentage (at.%)		
	YC500	YC1000	YC2000
-NH-COO- (B.E. = 531.9–532.1 eV)	53	88 (BE = 532.4 eV)	76
C=O (B.E. = 532.6–532.7 eV)	2	-	7
C-O (B.E. = 533.8–533.9 eV)	45	12	22

**Table 8:** Binding energies and percentages of C1s species on the surfaces of PUs made with polycarbonates of different molecular weights. XPS experiments.

Species	Percentage (at.%)		
	YC500	YC1000	YC2000
C-C, C-H (B.E. = 284.9 eV)	70	84	82
C-O (B.E. = 285.6–285.7 eV)	7	6	7
C-N (B.E. = 286.7–286.8 eV)	16	7	6
C=O (B.E. = 289.1–289.4 eV)	4	2	4
O-(C=O)-O (B.E. = 290.7–290.8 eV)	3	1	1

**Table 9:** Temperatures and weight losses of the thermal degradations of PUs made with polycarbonates of different molecular weights. DTGA experiments.

PU	1st Degradation		2nd Degradation		3rd Degradation	
	T_1_ (°C)	Weight Loss_1_ (%)	T_2_ (°C)	Weight Loss_2_ (%)	T_3_ (°C)	Weight Loss_3_ (%)
YC500	285	21	321	73	415	6
YC1000	292	30	311	60	412	10
YC2000	293	26	320	66	421	8

**Table 10:** Values of tan delta and temperature of the maximum of tan delta for PUs made with polycarbonates of different molecular weights. DMA experiments.

PU	tan delta	T_tan delta_ (°C)
YC500	0.99	39
YC1000	0.38	10
YC2000	0.16	17

**Table 11:** Parameters obtained from the stress–strain curves of PUs made with polycarbonates of different molecular weights.

PU	Young Modulus (kPa)	Yield Point		Break Point	
		σ_y_ (kPa)	ε_y_ (%)	σ_b_ (kPa)	ε_b_ (%)
YC500	30	-	-	2540	1083
YC1000	60	-	-	590	˃1119
YC2000	1800	8000	12	5000	119

## Data Availability

The original contributions presented in the study are included in the article/Supplementary Materials, further inquiries can be directed to the corresponding author.

## Supplementary Materials

The following supporting information can be downloaded at: https://www.mdpi.com/article/10.3390/polym16192724/s1, Figure S1: DSC curves of PUs made with polycarbonates of different molecular weights. Second heating run; Figure S2: TGA curves of PUs made with polycarbonates of different molecular weights; Table S1: Main diffraction peaks of the PUs made with polycarbonates of different molecular weights. X-ray diffraction experiments.

## References

[1] Ates M., Karadag S., Eker A.A., Eker B. (2022). Polyurethane foam materials and their industrial applications. Polym. Int..

[2] Kaur R., Singh P., Tanwar S., Varshney G., Yadav S. (2022). Assessment of bio-based polyurethanes: Perspective on applications and bio-degradation. Macromol.

[3] Lambert S., Wagner M. (2017). Environmental performance of bio-based and biodegradable plastics: The road ahead. Chem. Soc. Rev..

[4] Chen J., Gao Y., Shi L., Yu W., Sun Z., Zhou Y., Liu S., Mao H., Zhang D., Lu T. (2022). Phase-locked constructing dynamic supramolecular ionic conductive elastomers with superior toughness, autonomous self-healing and recyclability. Nat. Commun..

[5] Lee W.J., Oh H.G., Cha S.H. (2021). A brief review of self-healing polyurethane based on dynamic chemistry. Macromol. Res..

[6] Arévalo-Alquichire S., Morales-Gonzalez M., Diaz L.E., Valero M.F. (2018). Surface response methodology-based mixture design to study the influence of polyol blend composition on polyurethanes’ properties. Molecules.

[7] Brzeska J., Elert A.M., Morawska M., Sikorska W., Kowalczuk M., Rutkowska M. (2018). Branched polyurethanes based on synthetic polyhydroxybutyrate with tunable structure and properties. Polymers.

[8] Klinedinst D.B., Yilgör I., Yilgör E., Zhang M., Wilkes G.L. (2012). The effect of varying soft and hard segment length on the structure–property relationships of segmented polyurethanes based on a linear symmetric diisocyanate, 1, 4-butanediol and PTMO soft segments. Polymer.

[9] Guazzini T., Bronco S., Carignani E., Pizzanelli S. (2019). Tunable ionization degree in cationic polyurethanes and effects on phase separation. Eur. Polym. J..

[10] Gaymans R.J. (2011). Segmented copolymers with monodisperse crystallizable hard segments: Novel semi-crystalline materials. Prog. Polym. Sci..

[11] Xu Y., Petrovic Z., Das S., Wilkes G.L. (2008). Morphology and properties of thermoplastic polyurethanes with dangling chains in ricinoleate-based soft segments. Polymer.

[12] Samuels S.L., Wilkes G.L. (1973). The rheo-optical and mechanical behavior of a systematic series of hard-soft segmented urethanes. J. Polym. Sci. Polym. Symp..

[13] Petrović Z.S., Ferguson J. (1991). Polyurethane elastomers. Prog. Polym. Sci..

[14] Yu K., Xin A., Du H., Li Y., Wang Q. (2019). Additive manufacturing of self-healing elastomers. NPG Asia Materials.

[15] Liu Y., Li Z., Liu R., Liang Z., Yang J., Zhang R., Zhou Z., Nie Y. (2019). Design of self-healing rubber by introducing ionic interaction to construct a network composed of ionic and covalent cross-linking. Ind. Eng. Chem. Res..

[16] Liang Z., Huang D., Zhao L., Nie Y., Zhou Z., Hao T., Li S. (2021). Self-healing polyurethane elastomer based on molecular design: Combination of reversible hydrogen bonds and high segment mobility. J. Inorg. Organomet. Polym. Mater..

[17] Cho J.W., Jung Y.C., Chung Y.C., Chun B.C. (2004). Improved mechanical properties of shape-memory polyurethane block copolymers through the control of the soft-segment arrangement. J. Appl. Polym. Sci..

[18] Harris R.F., Joseph M.D., Davidson C., Deporter C.D., Dais V.A. (1990). Polyurethane elastomers based on molecular weight advanced poly (ethylene ether carbonate) diols. I. Comparison to commercial diols. J. Appl. Polym. Sci..

[19] Harris R.F., Joseph M.D., Davidson C., Deporter C.D., Dais V.A. (1990). Polyurethane elastomers based on molecular weight advanced poly (ethylene ether carbonate) diols. II. Effects of variations in hard segment concentration. J. Appl. Polym. Sci..

[20] Gunatillake P.A., Meijs G.F., McCarthy S.J., Adhikari R., Sherriff N. (1998). Synthesis and characterization of a series of poly (alkylene carbonate) macrodiols and the effect of their structure on the properties of polyurethanes. J. Appl. Polym. Sci..

[21] Tanaka H., Kunimura M. (2002). Mechanical properties of thermoplastic polyurethanes containing aliphatic polycarbonate soft segments with different chemical structures. Polym. Eng. Sci..

[22] Kojio K., Nonaka Y., Masubuchi T., Furukawa M. (2004). Effect of the composition ratio of copolymerized poly (carbonate) glycol on the microphase-separated structures and mechanical properties of polyurethane elastomers. J. Polym. Sci. B Polym. Phys..

[23] Furukawa M. (1994). Property-structure relationships of polyurethane elastomers: Improvement of hydrolytic stability and thermal stability. Appl. Polym. Symp..

[24] Pinchuk L. (1995). A review of the biostability and carcinogenicity of polyurethanes in medicine and the new generation of ‘biostable’ polyurethanes. J. Biomater. Sci. Polym. Ed..

[25] Tanzi M.C., Mantovani D., Petrini P., Guidoin R., Laroche G. (1997). Chemical stability of polyether urethanes versus polycarbonate urethanes. J. Biomed. Mater. Res..

[26] Kuran W., Sobczak M., Listos T., Debek C., Florjanczyk Z. (2000). New route to oligocarbonate diols suitable for the synthesis of polyurethane elastomers. Polymer.

[27] Christenson E.M., Anderson J.M., Hiltner A. (2004). Oxidative mechanisms of poly (carbonate urethane) and poly (ether urethane) biodegradation: In vivo and in vitro correlations. J. Biomed. Mater. Res. A.

[28] Wiggins M.J., MacEwan M., Anderson J.M., Hiltner A. (2004). Effect of soft-segment chemistry on polyurethane biostability during in vitro fatigue loading. J. Biomed. Mater. Res. Part. A.

[29] Khan I., Smith N., Jones E., Finch D.S., Cameron R.E. (2005). Analysis and evaluation of a biomedical polycarbonate urethane tested in an in vitro study and an ovine arthroplasty model. Part I: Materials selection and evaluation. Biomaterials.

[30] Khan I., Smith N., Jones E., Finch D.S., Cameron R.E. (2005). Analysis and evaluation of a biomedical polycarbonate urethane tested in an in vitro study and an ovine arthroplasty model. Part II: In vivo investigation. Biomaterials.

[31] Tang Y.W., Labow R.S., Revenko I., Santerre J.P. (2002). Influence of surface morphology and chemistry on the enzyme catalyzed biodegradation of polycarbonate-urethanes. J. Biomater. Sci. Polym. Ed..

[32] Liu N., Zhao Y., Kang M., Wang J., Wang X., Feng Y., Yin N., Li Q. (2015). The effects of the molecular weight and structure of polycarbonatediols on the properties of waterborne polyurethanes. Prog. Org. Coat..

[33] García-Pacios V., Jofre-Reche J.A., Costa V., Colera M., Martín-Martínez J.M. (2013). Coatings prepared from waterborne polyurethane dispersions obtained with polycarbonates of 1, 6-hexanediol of different molecular weights. Prog. Org. Coat..

[34] Eceiza A., Martin M.D., De La Caba K., Kortaberria G., Gabilondo N., Corcuera M.A., Mondragon I. (2008). Thermoplastic polyurethane elastomers based on polycarbonate diols with different soft segment molecular weight and chemical structure: Mechanical and thermal properties. Polym. Eng. Sci..

[35] Ha Y.M., Kim Y.O., Ahn S., Lee S.K., Lee J.S., Park M., Chung J.W., Jung Y.C. (2019). Robust and stretchable self-healing polyurethane based on polycarbonate diol with different soft-segment molecular weight for flexible devices. Eur. Polym. J..

[36] Paez-Amieva Y., Martín-Martínez J.M. (2024). Dynamic non-covalent exchange intrinsic self-healing at 20 °C mechanism of polyurethane induced by interactions among polycarbonate soft segments. Polymers.

[37] Paez-Amieva Y., Mateo-Oliveras N., Martín-Martínez J.M. (2024). Polyurethanes synthesized with blends of polyester and polycarbonate polyols—New evidences supporting the dynamic non-covalent exchange mechanism of intrinsic self-healing at 20 °C. Polymers.

[38] Paez-Amieva Y., Carpena-Montesinos J., Martín-Martínez J.M. (2023). Innovative device and procedure for in situ quantification of the self-healing ability and kinetics of self-healing of polymeric materials. Polymers.

[39] Ramakrishna S., Kumar K.S., Mathew D., Nair C.R. (2015). Long-living, stress-and pH-tolerant superhydrophobic silica particles via fast and efficient urethane chemistry; facile preparation of self-recoverable SH coatings. J. Mater. Chem. A.

[40] Paez-Amieva Y., Martín-Martínez J.M. (2023). Understanding the interactions between soft segments in polyurethanes: Structural synergies in blends of polyester and polycarbonate diol polyols. Polymers.

[41] Princi E., Vicini S., Castro K., Capitani D., Proietti N., Mannina L. (2009). On the micro-phase separation in waterborne polyurethanes. Macromol. Chem. Phys..

[42] Fuensanta M., Khoshnood A., Martín-Martínez J.M. (2020). Structure–properties relationship in waterborne poly (urethane-urea)s synthesized with dimethylolpropionic acid (DMPA) internal emulsifier added before, during and after prepolymer formation. Polymers..

[43] Mishra A.K., Chattopadhyay D.K., Sreedhar B., Raju K.V.S.N. (2006). FT-IR and XPS studies of polyurethane-urea-imide coatings. Prog. Org. Coat..

[44] Tardio S., Abel M.-L., Carr R.H., Watts J.F. (2019). The interfacial interaction between isocyanate and stainless steel. Int. J. Adhes. Adhes..

[45] Niemczyk A., Piegat A., Olalla Á.S., El Fray M. (2017). New approach to evaluate microphase separation in segmented polyurethanes containing carbonate macrodiol. Eur. Polym. J..

