# Supplementary material for: Influence of the Molecular Weight of the Polycarbonate Polyol on the Intrinsic Self-Healing at 20 °C of Polyurethanes
Source: Polymers (Basel). 2024 Sep 26;16(19):2724. doi: 10.3390/polym16192724 (PMC11478995; doi:10.3390/polym16192724)
Supplement: Supplementary file 1 [file polymers-16-02724-s001.zip › polymers-3197409-supplementary.pdf]

Influence of the molecular weight of the polycarbonate polyol on the intrinsic self-healing at 20 ºC of polyurethanes

Yuliet Paez-Amieva, José Miguel Martín-Martínez

Supplementary material

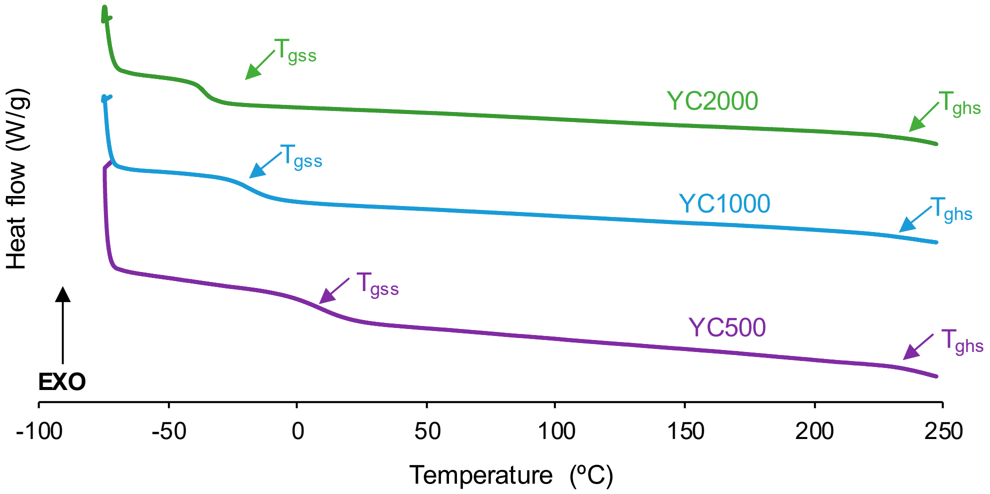

Figure S1. DSC curves of PUs made with polycarbonates of different molecular weights. Second heating run.

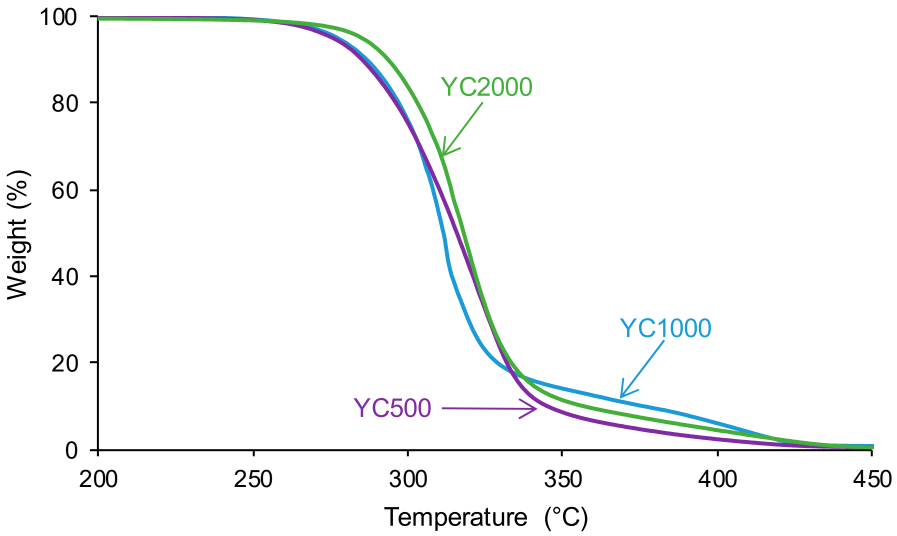

Figure S2. TGA curves of PUs made with polycarbonates of different molecular weights.

Table S1. Main diffraction peaks of the PUs made with polycarbonates of different molecular weights. X-ray diffraction experiments.

PU	2θ (°)	Intensity (a.u.)	2θ (°)	Intensity (a.u.)
YC500	19.9	3114	-	-
YC1000	20.0	1516	23.3	1763
YC2000	20.1	8438	23.3	4951
